# Relationship Between Heart Rate, Oxygen Consumption, and Energy Expenditure in Futsal

**DOI:** 10.3389/fpsyg.2021.698622

**Published:** 2021-08-11

**Authors:** Henrique Santos da Silva, Fabio Yuzo Nakamura, Marcelo Papoti, Alexsandro Santos da Silva, Julio Wilson Dos-Santos

**Affiliations:** ^1^Research Group on Exercise Physiology Applied to Sports Training (FITES), School of Science, São Paulo State University (UNESP), Bauru, Brazil; ^2^Post-Graduate Program in Motricity Sciences, São Paulo State University (UNESP), Bauru, Brazil; ^3^Research Center in Sports Sciences, Health Sciences and Human Development, CIDESD, University Institute of Maia, ISMAI, Maia, Portugal; ^4^School of Physical Education and Sport of Ribeirão Preto, University of São Paulo, Ribeirão Preto, Brazil; ^5^School of Science, Department of Physical Education, São Paulo State University (UNESP), Bauru, Brazil

**Keywords:** energy cost, metabolic equivalent, metabolic pathway, team sport, futsal match

## Abstract

The primary aim of this study was to compare the measured oxygen consumption (Measured*-V*O_2_) in a simulated futsal game (S-Game) with the estimated oxygen consumption (Estimated*-V*O_2_) through a regression equation between heart rate (HR) and oxygen consumption (*V*O_2_) (HR-*V*O_2_) in treadmill running, and a secondary aim was to calculate the total energy expenditure (EE) in S-Game. Ten professional players (22.20 ± 3.22 years) were evaluated. HR-*V*O_2_ was determined individually in the continuous test on the treadmill (Cont_Test_). The Measured*-V*O_2_ in S-Game was compared with the Estimated*-V*O_2_ in the Cont_Test_. Alactic and lactic pathways were estimated by *V*O_2_. The Estimated*-V*O_2_ presented no statistically significant difference with the Measured*-V*O_2_, using the paired *t*-test (*p* = 0.38). However, the correlation between Estimated- and Measured-*V*O_2_ was very weak (*r* = −0.05), and it presented poor agreement (concordance correlation coefficient = −0.04). In addition, a Bland–Altman plot presented bias of −2.8 ml/kg/min and individual difference as large as 19 ml/kg/min. The HR-*V*O_2_ determined by the Cont_Test_ was not a good individual predictor of *V*O_2_. The high intensity and intermittent nature of the futsal game possibly caused dissociation in the HR-*V*O_2_ relationship. Cont_Test_ is not recommended for estimating *V*O_2_ and calculating individual EE in the futsal game. This is recommended only for the group mean. The total EE in S-Game was 13.10 ± 1.25 kcal.min^−1^ (10.81 ± 1.57 metabolic equivalents). The contributions from the metabolic pathways were as follows: aerobic (93%), alactic (5%), and lactic (2%).

## Introduction

The linearity of the relationship between heart rate (HR) and oxygen consumption (*V*O_2_) (HR-*V*O_2_) is observed in progressive continuous exercise (Achten and Jeukendrup, 2003). From the regression equation obtained in treadmill running, it is possible to estimate *V*O_2_ in team sports (Esposito et al., 2004; Castagna et al., 2007) and, consequently, the energy expenditure (EE) (Rodrigues et al., 2011; Makaje et al., 2012; Beato et al., 2016). However, the thermal and emotional stress and the dehydration of the competition can elevate the HR without affecting the *V*O_2_, changing the linearity of the HR-*V*O_2_ (Esposito et al., 2004; Bangsbo et al., 2006), and inducing an estimation error (Achten and Jeukendrup, 2003; Buchheit et al., 2009). Despite that, HR-*V*O_2_ has been accepted and proposed to estimate *V*O_2_ in intermittent sports, such as soccer (Esposito et al., 2004) and futsal (Castagna et al., 2007). However, Buchheit et al. (2009) do not recommend the use of HR-*V*O_2_ to estimate *V*O_2_ in the handball game, since the estimated *V*O_2_ was lower than the *V*O_2_ measured in the game.

Concerning futsal, a team sport characterized by the intermittent actions of high intensity, accelerations, decelerations, and changes of direction (Makaje et al., 2012), the average intensity of game resulting in 90% maximum HR (Castagna et al., 2009), and blood lactate ([La^−^]) can reach 8.3 mmol/L (Dos-Santos et al., 2020), the HR-*V*O_2_ to predict game-specific *V*O_2_ is still not clear. In addition, HR-*V*O_2_ estimates only EE from the aerobic pathway, without considering the lactic and alactic anaerobic pathways. The contribution of the alactic anaerobic pathway from adenosine triphosphate–creatine phosphate (ATP–CP) can be made by calculating the fast component of excess postexercise oxygen consumption (EPOC) (Margaria et al., 1933; Beneke et al., 2002; Bertuzzi et al., 2007), while the EE of the lactic anaerobic pathway can be estimated through the O_2_ equivalent for [La^−^] (Di Prampero and Ferretti, 1999). These two procedures have been adopted to estimate EE from the anaerobic pathways and to calculate total EE in different sports, such as in tae kwon do, climbing, rowing, and table tennis (Bertuzzi et al., 2007; de Campos Mello et al., 2009; Campos et al., 2012; Zagatto et al., 2016). However, EE from the aerobic pathway has not yet been used in the futsal game.

To our knowledge, there are as yet no studies investigating total EE in futsal, considering the three energetic pathways. Knowing the total EE allows better planning of diet and training. Thus, the primary objective of this study was to compare measured oxygen consumption (Measured*-V*O_2_) in a simulated futsal game (S-Game) with the estimated oxygen consumption (Estimated*-V*O_2_) through a regression equation between HR and *V*O_2_ obtained in the continuous test on the treadmill (Cont_Test_), and the secondary objective was to determine the total EE in S-Game. Considering the intermittent characteristic and the high intensity of the futsal game, it is expected that the *V*O_2_ estimated through the regression equation between HR and *V*O_2_ in the continuous test does not present good agreement with the *V*O_2_ measured in the game. We hypothesized that the Estimated*-V*O_2_ would not correspond to the Measured*-V*O_2_ in S-Game.

## Materials and Methods

### Subjects

Ten professional futsal players of a team participated in the study (22.2 ± 3.2 years; 178 ± 6 cm; 70.2 ± 9.7 kg and 11.8 ± 4.5% of fat), all of them with a minimum experience of 5 years in futsal training and competition, i.e., daily training in two periods (3–4 h/day), 5–6 days/week. Players belonged to a team that played in the main competition in the State of São Paulo, Brazil, the Paulista League. Participants were previously informed of all procedures and signed a consent form. The design and protocol of the study conformed to the ethical standards established in the Declaration of Helsinki (2013) and was approved by the Ethics Committee of the University, according to the national laws (CAAE: 41515915.5.0000.5398).

### Proceedings

The characteristics of the subjects were made by measuring height [using stadiometer (Welmy, Santa Bárbara do Oeste, Sã~o Paulo, Brazil)], mass, and body composition [using the dual-energy X-ray absorptiometry (DXA), Discovery Wi, Hologic, Bedford, MA, USA], adopting all the procedures of the manufacturer. A Cont_Test_ was performed to obtain a linear regression equation (*y* = *ax* + *b*) and to calculate the Estimated*-V*O_2_ in S-Game. In addition, HR-*V*O_2_ was determined for each player in S-Game to analyze the slope of the linear equation and to compare it with that generated by Cont_Test_. The Estimated*-V*O_2_ was compared with the Measured*-V*O_2_. The EE corresponding to each metabolic pathway (i.e., aerobic, alactic, and lactic anaerobic) was determined in S-Game. All tests were performed in random order between 9:00 a.m. and 12:00 p.m., without any vigorous physical exertion in the previous 24 h. At least a 48-h interval was interspersed in the Cont_Test_ and S-Game for each player. The ambient temperature in all assessments varied between 28 and 32°C.

### Continuous Test on the Treadmill (Cont_Test_) and Simulated Futsal Game (S-Game)

The Cont_Test_ was performed on a treadmill (Inbramed ATL, Porto Alegre, Brazil) with a slope of 1%, an initial velocity of 8.0 km/h, and an increase of 1.0 km/h at each minute, until exhaustion (Kuipers et al., 2003). The *V*O_2max_ was determined according to the criteria proposed by Howley et al. (1995). Players from the same team participated in the S-Game, which consisted of four outfield players and the goalkeeper, randomly selected, following the rules of futsal on the court measuring 40 × 20 m. The data collection time for each player in S-Game was 10 min, since substitutions in futsal are unlimited, and in official matches, the players stay an average of 10 min playing effectively on the court before being substituted (Castagna et al., 2009; Makaje et al., 2012). In addition, a 10-min effort period has been used in the studies with S-Game (Castagna et al., 2009; Milioni et al., 2016). Considering the time taken for the data collection, the use of a portable gas analyzer, and the availability of players by the technical committee of the team, the data collection of S-Game was performed with one player in each 10-min S-Game, on separated days by more than 48 h. The physical coach of the team refereed the games, and the head coach was present to guide and encourage the players. Before the S-Game, a blood sample (i.e., 25 μl of the earlobe) was taken from the players for the analysis of [La^−^] at rest, and after blood collection, *V*O_2_ was measured at rest for 10 min in a sitting position. Subsequently, the players performed a standardized 10-min warm-up (e.g., jogging–running free = 2 min, dynamic exercises = 3 min, and exercises with ball = 5 min), and during the 10-min S-Game, the *V*O_2_ and HR were also measured. After the S-Game, *V*O_2_ was measured at rest during 10 min for the EPOC examination, and the blood samples were taken at 1, 3, 5, and 7 min to determine [La^−^] peak.

### Measurements of the Physiological Parameters

In all procedures, the HR was recorded for each second. The maximum HR (HR_max_) was considered the highest value in Cont_Test_. The gas analyses were performed with the K4 b2 device (Cosmed Srl, Rome, Italy), with all calibrations and other procedures recommended by the manufacturer. The respiratory variables were smoothed every five points and interpolated every second to reduce the artifacts (Özyener et al., 2001). Estimated*-V*O_2_ was calculated by HR-*V*O_2_ for each player in Cont_Test_, obtaining a linear regression equation (*y* = *ax* + *b*), and considering the mean of HR (HR_mean_) obtained in S-Game. Measured*-V*O_2_ was calculated by the area of *V*O_2_ by each HR record, disregarding the *V*O_2_ at rest, to determine EE only during futsal game.

Samples of [La^−^] were collected in capillary tubes (i.e., 50 μl of 1% sodium fluoride) and analyzed on YSI 2300 SPORTS (Yellow Springs Instruments, Yellow Springs, OH, USA). The energy demand of the aerobic (*W*_AER_), alactic anaerobic (*W*_PCR_), and lactic anaerobic (*W*_[La_-_]_) metabolism was estimated as follows: *W*_AER_ = difference between the area of *V*O_2_ during the S-Game (i.e., calculated by the trapezoidal integration method) of the *V*O_2_ at rest (i.e., calculated by the product between the *V*O_2_ at rest and the total duration of the game) (Beneke et al., 2002; Bertuzzi et al., 2007; Campos et al., 2012); *W*_PCR_ = fast component of EPOC measured after S-Game, i.e., calculated by the product between the amplitude and tau (τ) obtained by means of the bi-exponential adjustment of *V*O_2_ (Beneke et al., 2002; Bertuzzi et al., 2010); and *W*_[La_-_]_ = difference between the peak lactate of the S-Game and at rest (Δ [La^−^]), considering the equivalent energy of 3 ml of O_2_·per kg for each 1 mmol/L of [La^−^] (Di Prampero and Ferretti, 1999). Thus, the total EE (i.e., *W*_AER_ + *W*_PCR_ + *W*_[La_-_]_) was expressed in several ways to facilitate its application, considering the equivalent for O_2_ ml/kg/min in kilocalories (kcal) and metabolic equivalent (MET) (Bertuzzi et al., 2007; McArdle et al., 2014).

### Statistical Analysis

The Shapiro–Wilk test was used to verify the normality of the data. After confirming the normality of the data, the paired *t*-test was applied. The 95% CI was determined, and the effect size (ES) was calculated by using Cohen's *d* (Cohen, 1988), considering ES: <0.19 trivial, 0.20–0.49 small, 0.50–0.79 moderate, and >0.80 large. The Pearson's correlation was determined and classified according to the values of *r* as follows: very weak (0.0–0.2), weak (0.2–0.4), moderate (0.4–0.7), strong (0.7–0.9), and very strong (0.9–1.0) (Glickman and Rowntree, 1982). The Bland–Altman analysis was used to verify the agreement between Measured*-V*O_2_ and Estimated*-V*O_2_, as well as the Lin's concordance correlation coefficient (CCC), following the scale for strength of agreement: poor (<0.90), moderate (0.90–0.95), substantial (0.95–0.99), and almost perfect (>0.99) (Lin, 1989). The level of significance was set at 5%. The calculations were performed using the following statistical programs: IBM SPSS Statistics software for Windows, version 22.0 (IBM Corp, Armonk, NY, USA), and MedCalc 9.2 (MedCalc Software bv, Ostend, Belgium).

## Results

**Figure 1:**
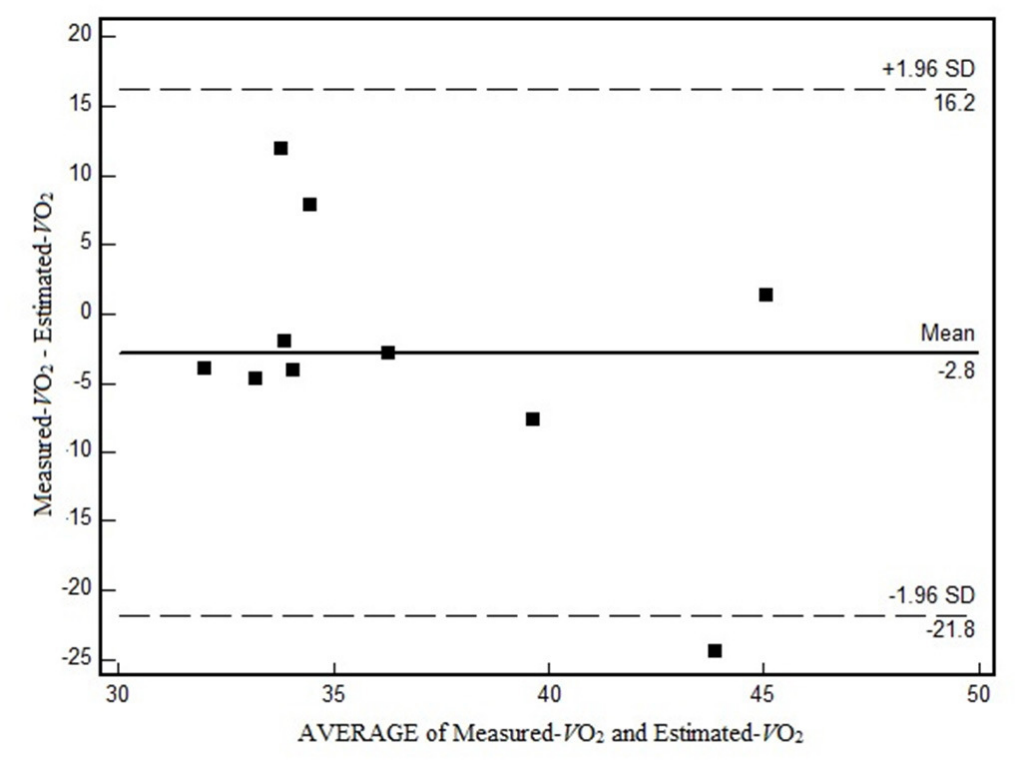
The Bland–Altman analysis for oxygen consumption (*V*O_2_ ml/kg/min) between Measured*-V*O_2_ and Estimated*-V*O_2_.

**Figure 2:**
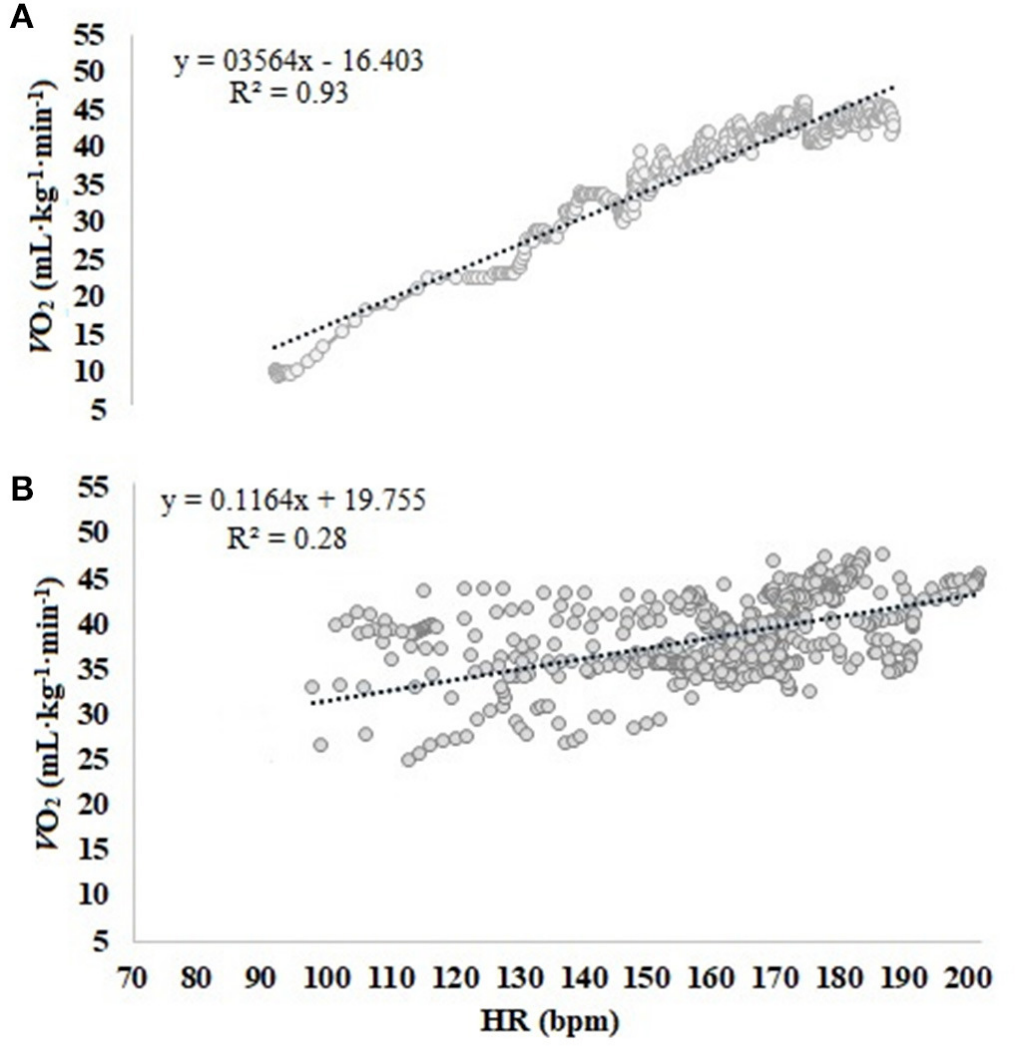
Relationship between heart rate (HR, bpm) and oxygen consumption (*V*O_2_, ml/kg/min) of an athlete, during the tests: **(A)** continuous treadmill test (Cont_Test_) and **(B)** simulated futsal game (S-Game).

The values of Measured*-V*O_2_ and Estimated*-V*O_2_ presented no significant difference (Table 1). Considering only the *W*_AER_, the EE also did not differ statistically (i.e., measured = 12.19 ± 1.09 and estimated = 13.32 ± 3.17). However, the correlations demonstrated that Estimated*-V*O_2_ did not correspond to Measured*-V*O_2_ individually (Table 1). In the Bland–Altman analysis (Figure 1), it is possible to observe the data scatter and the low agreement between the Estimated*-V*O_2_ and Measured*-V*O_2_, a bias of −2.8 ml/kg/min, and the limits of agreement varying in 19 ml/kg/min. When comparing the slope averages of the linear regression equation generated by the HR-*V*O_2_ in the Cont_Test_ (0.48 ± 0.12) and S-Game (0.28 ± 0.21), significant differences were observed between the slopes (*p* = 0.007). The correlation coefficients of the HR-*V*O_2_ in Cont_Test_, *r* = 0.95 ± 0.03, and in S-Game, *r* = 0.61 ± 0.27, presented significant difference (*p* = 0.003), and the explication coefficients were *r*^2^ = 0.91 ± 0.05 and *r*^2^ = 0.44 ± 0.29, respectively. The HR-*V*O_2_ of a player in the two situations (i.e., Cont_Test_ and S-Game) allows visualizing these differences (Figure 2). The S-Game presented a high-intensity level as expected for futsal as follows: HR_mean_ = 163 ± 7 bpm, corresponding to HR_max_ = 90 ± 4%. The HR_max_ in S-Game was 188 ± 11 bpm, whereas it was 182 ± 7 bpm in Cont_Test_. [La^−^] peak after S-Game was 3.57 ± 1.34 mmol/L.

**Table 1:** Comparison between the measured oxygen consumption (Measured*-V*O_2_) in simulated futsal game (S-Game) and the estimated oxygen consumption (Estimated*-V*O_2_) estimated by the HR-*V*O_2_ regression equation from the continuous treadmill test.

	**Measured-*V*O_**2**_**	**Estimated-*V*O_**2**_**	***p-*value**	**Cohen's ES**	**Pearson's**	**Lin's**
	**(95% CI)**	**(95% CI)**		**(*d*)**	**(*r*)**	**(CCC)**
*V*O_2_ (mL.Kg.min−1)	35.21 ± 4.90 (32.71–38.72)	38.04 ± 8.13 (32.23–43.86)	0.38	0.43 (small)	−0.05 (very weak)	−0.04 (poor)

*Values expressed as mean ± SD. HR, Heart rate; VO_2_, oxygen consumption; ES, effect size; 95% CI lower and upper limits*.

Table 2 shows the total EE and the proportions of the contribution of each energy pathway in the S-Game, disregarding the resting values of each individual. The anaerobic EE represented 7% of the total contribution in S-Game.

**Table 2:** The energy expenditure of the aerobic (*W*_AER_), alactic anaerobic (*W*_PCR_), and lactic anaerobic (*W*_[La_-_]_) metabolism in S-Game.

	***W*_**AER**_**	***W*_**PCR**_**	***W*_**[La**_-_**]**_**	**Total**
*V*O_2_ (ml/kg/min)	35.21 ± 4.90	1.92 ± 0.83	0.70 ± 0.39	37.83 ± 5.49
*V*O_2_ (L/min)	2.44 ± 0.22	0.13 ± 0.05	0.05 ± 0.03	2.62 ± 0.25
EE (kcal.min^−1^)	12.19 ± 1.09	0.66 ± 0.25	0.25 ± 0.14	13.10 ± 1.25
EE (METs)	10.06 ± 1.40	0.55 ± 0.24	0.20 ± 0.11	10.81 ± 1.57
Percentage (%)	93.15 ± 2.29	4.99 ± 1.70	1.87 ± 1.04	100.00 ± 0.00

*VO_2_, oxygen consumption; EE, energy expenditure; METs, metabolic equivalents*.

## Discussion

The primary objective of this study was to compare the Measured*-V*O_2_ with the Estimated*-V*O_2_ in futsal, through the regression equations generated from the HR-*V*O_2_ obtained in the Cont_Test_. Although the Estimated*-V*O_2_ presented no significant difference with the Measured*-V*O_2_, they presented a very weak correlation and poor agreement (Table 1). It could be remarked that, according to this main finding, the HR-*V*O_2_ determined by the Cont_Test_ was not a good individual predictor of *V*O_2_. Considering the secondary objective, the aerobic energy demand in S-Game was 93%, anaerobic alactic, 5%, and anaerobic lactic, 2%.

There was no significant difference (using the paired *t*-test) between the Measured-*V*O_2_ and the Estimated-*V*O_2_, indicating that our hypothesis should be refuted (i.e., HR-*V*O_2_ of the Cont_Test_ would not be a good game-related *V*O_2_ predictor). However, the correlation and concordance tests evidenced the low predictive capacity of *V*O_2_ by HR-*V*O_2_ from a test on the treadmill (i.e., Cont_Test_) for estimating *V*O_2_ in futsal game. In addition, the correlation coefficients from Cont_Test_ and S-Game were different, *r* = 0.95 and *r* = 0.61, respectively. In futsal recreational game with high school students, the estimated and measured *V*O_2_ in futsal game did not differ either (Castagna et al., 2007). Although the authors pointed out the validity of the estimation of *V*O_2_ from the regression equation in treadmill running, they stated that HR might have a less predictive capacity of real aerobic involvement during intermittent activities compared with continuous exercise. Therefore, they recommended that the HR-*V*O_2_ be only used for groups and not individually since the correlation coefficient of the HR-*V*O_2_ presented a statistical difference (*p* < 0.001) between the futsal game (*r* = 0.83) and the treadmill running (*r* = 0.96). Those authors assumed that other occasional activities, such as high-speed running or isometric muscle actions, may explain some differences between HR and *V*O_2_ in intermittent exercise vs. continuous exercise. In addition, the heat and stress factors of the game can influence the dissociation of the response of HR-*V*O_2_ (Achten and Jeukendrup, 2003). Another interesting result that must be taken into account when analyzing the HR-*V*O_2_ is the intensity. Notably, in this study, the average HR was HR_max_ = 90%, similar to the official games (Barbero-Alvarez et al., 2008), while in the recreational game with high school students, the average HR was HR_max_ = 83% (Castagna et al., 2007), resulting in a weaker correlation in the HR-*V*O_2_ when the game is more intense (*r* = 0.61 vs. 0.83, respectively). However, the HR-*V*O_2_ from the treadmill test has been used to estimate the *V*O_2_ in team sports, such as soccer.

In soccer, Hoff et al. (2002) indicated HR as a valid indicator of *V*O_2_ in a 5 vs. 5 small-sided game and in a dribbling field test. However, the authors used average values for the correlation analysis between HR and *V*O_2_ and did not compare the correlation coefficients between the different situations. Esposito et al. (2004) did not observe a significant difference between the *V*O_2_ measured in the specific field test to soccer and that estimated by the equation in the treadmill test, recommending that the HR-*V*O_2_ regression equation obtained on the treadmill is valid to calculate *V*O_2_. In this study, Measured*-V*O_2_ and Estimated*-V*O_2_ by HR-*V*O_2_ were not different either. However, the results of the Estimated*-V*O_2_ by HR-*V*O_2_ did not present concordance. We compared the Estimated*-V*O_2_ by HR-*V*O_2_ with the Measured*-V*O_2_ in S-Game, which is more specific than a field test, as used in studies with soccer (Hoff et al., 2002; Esposito et al., 2004). Although in the study by Hoff et al. (2002), the subjects were also evaluated in a 5 vs. 5 small-sided soccer game, and the calculation of the individual *V*O_2_ using the HR-*V*O_2_ equation from the treadmill was not carried out (Hoff et al., 2002). Besides, futsal is more intense than soccer (Barbero-Alvarez et al., 2008), since the actions are carried out in a smaller space and with more frequent changes of direction. The intermittent characteristic and the high intensity of the futsal game certainly contributed to the difference observed between HR and *V*O_2_ of S-Game and one obtained in continuous exercise test (Figure 2). Considering different methods used in the studies, and although some authors recommended the use of the HR-*V*O_2_ in soccer to estimate the *V*O_2_, the methods applied in this study demonstrated that the individual HR-*V*O_2_ in continuous exercise in treadmill can estimate different *V*O_2_ values of the Measured*-V*O_2_ in futsal game for each player.

Contrary to the studies cited above, in a study with handball players, Buchheit et al. (2009) did not recommend the use of HR-*V*O_2_ to estimate *V*O_2_. The authors found a good HR-*V*O_2_ for the treadmill test of goodness of fit (*r*^2^ = 0.96) but a moderate HR-*V*O_2_ for the handball game and intermittent exercise (*r*^2^ = 0.63 and *r*^2^ = 0.58, respectively). Moreover, *V*O_2_ estimated from the HR-*V*O_2_ in the treadmill test was lower than the *V*O_2_ measured in the handball game (*p* = 0.03). It was also possible to observe a large difference between *V*O_2_ measured in a handball game and that estimated from the intermittent exercise, 8.7 and 11.6 ml/kg/min, respectively. In handball, there are accelerations, decelerations, jumps, changes of direction, and actions of the upper limbs that increase the active muscle mass, which differs a lot from running on the treadmill.

The very weak correlation between Estimated*-V*O_2_ and Measured*-V*O_2_ (*r* = −0.05), ES estimated by Cohen's *d*, classified as small (0.4), and the CCC as poor (CCC = −0.04) corroborate results observed in handball (Buchheit et al., 2009), making clear the difference between the characteristics of the continuous exercise with the intermittent sports. In our results (Figure 2), it is possible to observe the difference in the dispersion of the data between the two exercise situations evaluated. The difference found between the slopes of the regression lines, Cont_Test_ (0.48 ± 0.12) and S-Game (0.28 ± 0.21) (*p* = 0.007), is an indication that the HR-*V*O_2_ responded differently in both Cont_Test_ and S-Game. A Bland–Altman plot in this study presented a bias of −2.8 ml/kg/min and individual difference as large as 19 ml/kg/min. The result of bias was similar to those from another study on futsal, −2.2 ml/kg/min, while the limit of agreement in this study was higher than 8 ml/kg/min (Castagna et al., 2007), however, the higher limit of agreement, in comparison with the results of Castagna et al. (2007), 8 ml/kg/min. The higher intensity of the S-Game in this study (90% HR_max_) compared with 83% HR_max_ (Castagna et al., 2007) can explain the difference between the two studies. As a result, the higher the intensity of the intermittent exercise, the greater the dissociation in HR-*V*O_2_, reinforcing the data from Balsom et al. (1992), which suggested that HR increased disproportionately to the *V*O_2_ after sprinting activities.

The low correlation and the agreement between Measured*-V*O_2_ and Estimated*-V*O_2_ of this study indicate that the equations of HR-*V*O_2_ generated from the continuous test are not good for estimating *V*O_2_ individually, and although there is no difference to estimate the “rough” *V*O_2_ of the group, it is not recommended to plan diets or the training load from this calculation, due to the biological individuality of each athlete and unpredictable situations in the game, which influence the HR-*V*O_2_.

The use of HR-*V*O_2_ in the estimation of *V*O_2_ in team sports should be viewed with caution since the literature presents contradictory results. Studies with soccer (Esposito et al., 2004) and futsal (Castagna et al., 2007) indicated the validity of HR-*V*O_2_, whereas in handball (Buchheit et al., 2009) and in this study with futsal, HR-*V*O_2_ was not valid for the estimation of *V*O_2_ by HR from the continuous progressive test. Another interesting fact in this study is that the comparison between Measured*-V*O_2_ and Estimated*-V*O_2_ from HR-*V*O_2_ was made by calculating the integral area of *V*O_2_ over the time during S-Game since for each recorded HR there are different *V*O_2_ values. In other studies, *V*O_2_ was estimated using the HR_mean_ of the exercise (Esposito et al., 2004; Castagna et al., 2007; Makaje et al., 2012), which can be a bias of those studies.

In addition to HR-*V*O_2_ and the aerobic EE, we also calculated the total EE (i.e., *W*_AER_ + *W*_PCR_ + *W*_[La_-_]_). The same method for calculating the total EE has been used in other sports, as tae kwon do (Campos et al., 2012), climbing (Bertuzzi et al., 2007), rowing (de Campos Mello et al., 2009), and table tennis (Zagatto et al., 2016). In all these studies about EE and as also verified in this study, the calculated value of the lactic anaerobic pathway has been the lowest value, which can be a characteristic of the sports evaluated or a limitation of the method. Certainly, the measurement of anaerobic EE is more difficult than aerobic EE, and it has limitations. For example, in an incremental exercise, it is recommended that stages between 3 and 6 min be used to obtain precise [La^−^] measurements (Bentley et al., 2007), ensuring the efflux of muscle lactate to not underestimate the [La^−^] value. In contrast, if after 5–6 min the lactate efflux from the muscle into the blood is guaranteed, the measurement taken in a time above this can lead to loss of information. Completing this idea, Stølen et al. (2005) pointed out that, in soccer, the [La^−^] measure depends largely on the activity pattern in 5 min preceding the sample collection, since [La^−^] results from the production/removal ratio, which is influenced by the value of the lactate produced, activity during the recovery period, and aerobic capacity. In addition, for the 5 vs. 5 small-sided games in soccer, Hoff et al. (2002) indicated 4 min of play to reach at least 3 min at high intensity. Thus, we suggested that in future studies, mainly on futsal, blood samples be taken with *V*O_2_ at 3, 4, and/or 5 min to verify if the length of the playing period can influence the calculation of the lactic EE per minute, since the intensity of the futsal game is higher than in soccer and HR average is 90% HR_max_ (Barbero-Alvarez et al., 2008), with less variation in HR (i.e., coefficient of variation = 7%) during the game (Dos-Santos et al., 2020).

The alactic EE also has limitations and can be underestimated due to the intermittent character of the futsal game with changes between high-intensity activity and the activities of lower intensity and pauses, which reduce the *V*O_2_. However, the *V*O_2_ recovery of those *V*O_2_ reduction periods is not considered or measured during the game, limiting the exact calculation of the *W*_PCR_.

The EE in the S-Game was determined, disregarding the resting *V*O_2_, to account only the activity EE of futsal. The total EE measured was 13.10 ± 1.25 kcal.min^−1^, *W*_AER_ = 93%, *W*_PCR_ = 5%, and *W*_[La_-_]_ = 2%. The aerobic EE measured was 12.19 ± 1.09 kcal.min^−1^. In futsal game with recreational players, Beato et al. (2016) found 634 ± 92 kcal for 52 ± 2 min, (≈12.19 kcal.min^−1^), and Makaje et al. (2012) found 595 ± 50 kcal (≈14.81 kcal.min^−1^) for elite players and 543 ± 67 kcal (≈13.57 kcal.min^−1^) for amateurs. Both studies were estimated by HR-*V*O_2_ relation, and they were close to the values evidenced in this study. However, in official games, Rodrigues et al. (2011) found higher values than the ones in the present study and the other studies, 18 kcal.min^−1^. In this case, the EE is expected to be higher in official games, or the value might have been overestimated, reinforcing that caution is needed in estimating EE from HR*-V*O_2_.

Considering the EE expressed in METs, the total EE of S-Game was 10.81 METs, which is a higher EE than in other team sports such as soccer (10.0 METs) and basketball (7.0 METs) (Ainsworth et al., 2000). The results expressed in METs of the EE of S-Game reinforce the findings of Barbero-Alvarez et al. (2008), which show that futsal is more intense than soccer, basketball, and handball. Our results can serve as a reference to guide and assist in assessment and prescription programs for weight control and exercise for health.

Although there is a limitation in the determination of the alactic and lactic anaerobic in intermittent exercise, it was possible to add information and to obtain the EE closest to the real one. This is the first study that proposed to investigate the anaerobic EE response and to add information about total EE in futsal. Further studies on total EE in futsal shorter-duration games are needed so that the anaerobic pathways are not thus underestimated. The shorter-duration games supposedly demand greater stress, intensity, heat production, intermittency, and alteration of game activities, i.e., the variables that may interfere with the physiological responses of the players.

## Conclusion

The HR-*V*O_2_ from the continuous test did not accurately estimate *V*O_2_ in the futsal game. HR-*V*O_2_ is not recommended for estimating *V*O_2_ and calculating individual EE in futsal, since it does not present acceptable agreement and correlation with the Measured*-V*O_2_ in the futsal game. The values estimated by HR-*V*O_2_ approach the average value of the game, which can only be used to estimate the “rough” *V*O_2_ of groups. The S-Game presented the total EE (i.e., *W*_AER_ + *W*_PCR_ + *W*_[La_-_]_) of 13.10 ± 1.25 kcal.min^−1^. In futsal (S-Game), the highest demand came from the aerobic pathway, 93%, from the alactic anaerobic pathway, 5%, and from the lactic anaerobic pathway, 2%.

## Data Availability Statement

The original contributions and raw data presented in the study are included in the article, further inquiries can be directed to the corresponding author.

## Ethics Statement

The studies involving human participants were reviewed and approved by Ethics Committee of School of Science, São Paulo State University (UNESP), Bauru, Brazil, in accordance with local laws and ethical standards established in the Declaration of Helsinki. The patients/participants provided their written informed consent to participate in this study.

## Author Contributions

HS and JD-S planed the study. HS contributed to the data collection, statistical analysis, and manuscript writing. AS contributed to the analysis and design. FN and MP have made substantial contributions to conception and design. JD-S, HS, and FN reviewed the manuscript, and JD-S contributed to conception and approval of the final version for publication. All authors have approved the final version of the manuscript.

## Conflict of Interest

The authors declare that the research was conducted in the absence of any commercial or financial relationships that could be construed as a potential conflict of interest.

## Publisher's Note

All claims expressed in this article are solely those of the authors and do not necessarily represent those of their affiliated organizations, or those of the publisher, the editors and the reviewers. Any product that may be evaluated in this article, or claim that may be made by its manufacturer, is not guaranteed or endorsed by the publisher.
